# German general practitioners’ experiences during the COVID-19 pandemic and how it affected their patient care: A qualitative study

**DOI:** 10.1080/13814788.2022.2156498

**Published:** 2023-02-01

**Authors:** Lisa Makowski, Hanna Schrader, Sandra Parisi, Jana Ehlers-Mondorf, Stefanie Joos, Hanna Kaduszkiewicz, Ildikó Gágyor

**Affiliations:** a Department of General Practice, University Hospital Wuerzburg, Wuerzburg, Germany; b Institute of General Practice, Kiel University, Kiel, Germany; c Institute for General Practice and Interprofessional Healthcare, University Hospital Tuebingen, Tuebingen, Germany

**Keywords:** COVID-19 pandemic, qualitative study, general practice, doctor-patient-relationship, team cohesion

## Abstract

**Background:**

General practitioners (GPs) play a significant role in providing medical care to patients affected by the coronavirus disease 2019 (COVID-19). Little is known about the impact of the pandemic on patient care from the perspective of GPs.

**Objectives:**

To gain insight into GPs’ experiences of the COVID-19 pandemic and its impact on patient care in Germany.

**Methods:**

From August to December 2020, qualitative, semi-structured telephone interviews were conducted with 22 GPs from four randomly selected federal states in Germany. We analysed the data according to Kuckartz’s method of content analysis.

**Results:**

Five themes emerged: changes in healthcare system, practice routines, patient care, personal life, and improving health crisis preparedness. Communication with authorities and following rapidly changing guidelines were the biggest challenges during the pandemic. Teamwork and collegial exchange in the practice were seen as important sources of support to overcome these barriers. Participants stated that they managed to secure care but expressed concerns about how social distancing might affect the doctor-patient relationship. In their professional and private lives, GPs perceived themselves as role models with a high responsibility for the health of others. Consistent guidance by health authorities and reliable information were raised as necessary for managing patient care in the pandemic.

**Conclusion:**

Findings show that patient care was successful but GPs’ adaptation to unprecedented conditions was limited by poor communication and collaboration with health authorities. Therefore, providing adequate support services by policymakers is essential to strengthen primary care in future health crises.

KEY MESSAGES

- In general practitioners’ view, patient care during the pandemic was restricted by poor communication and cooperation with health authorities.
- Teamwork and collegial exchange in group practices were perceived as vital support factors.
- General practitioners attributed high responsibility to their role as physicians during the pandemic.

## Introduction

Due to the outbreak of COVID-19, the healthcare system had to adapt to the current crisis quickly. Healthcare providers are on the frontline and at an increased risk regarding infection and mortality [1]. GPs play a key role in providing primary care for patients and are the first point of contact for information, advice and referral [2].

Several studies have already investigated the impact of the pandemic on GPs in other countries. It was shown that GPs faced enormous challenges during the pandemic, for example, poor communication among authorities, bureaucratic obstacles, a lack of public recognition of their contribution and the need to quickly restructure their daily practice to minimise the infection risk [3,4]. It was also reported that GPs had to deal with shortages of personal protective equipment (PPE), consultation decrease and financial losses [5]. To provide care to their patients, many GPs rapidly implemented remote consulting, though facing new challenges, such as difficulties assessing patients with complex conditions and challenges to the therapeutic relationship [6]. Furthermore, high levels of stress and anxiety, depressive symptoms and post-traumatic stress symptoms were found in GPs during the COVID-19 crisis [7].

In the German healthcare system, the Federal Ministry of Health develops healthcare policies at the federal level, and regional health departments regulate public health services and are responsible for protection against infections. In the pandemic, GPs played an important role in counselling their patients and were engaged in diagnosing and treating patients, testing potentially infectious patients, or monitoring patients in quarantine. German primary care is mainly represented by small, decentralised units, run by self-employed GPs, which might hinder an efficient response to the outbreak [8]. Due to the specific organisation of the healthcare system and primary care in Germany, international findings may not represent the pandemic-related experiences of German GPs.

Thus, this study aimed to investigate how German GPs experienced the first months of the COVID-19 pandemic. Notably, the potential impact of the crisis on the therapeutic relationship, emotional and professional support factors and GPs’ view on improving epidemic control measures should be explored more deeply. As the pandemic continues, GPs are constantly exposed to new challenges. Learning from the experiences of the first wave seems particularly important, especially regarding resources and support structures to help GPs in future health crises.

## Methods

### Study design

This qualitative study is part of a cross-sectional study with a mixed methods design. This paper presents the results of qualitative semi-structured interviews with GPs in Germany. Quantitative results are not presented in this article and are currently under review. Findings are reported following The Standards for Reporting Qualitative Research [9]. In total, 22 interviews were conducted with a mean length of 22 min. Interviews were conducted by telephone, given the nationwide COVID-19 restrictions. Participants were interviewed from 17 August 2020 until 11 December 2020, a period of a temporary decline of case numbers in Germany, followed by a steep increase in case of numbers in all federal states in Germany in autumn.

### Recruitment

**Figure 1.**
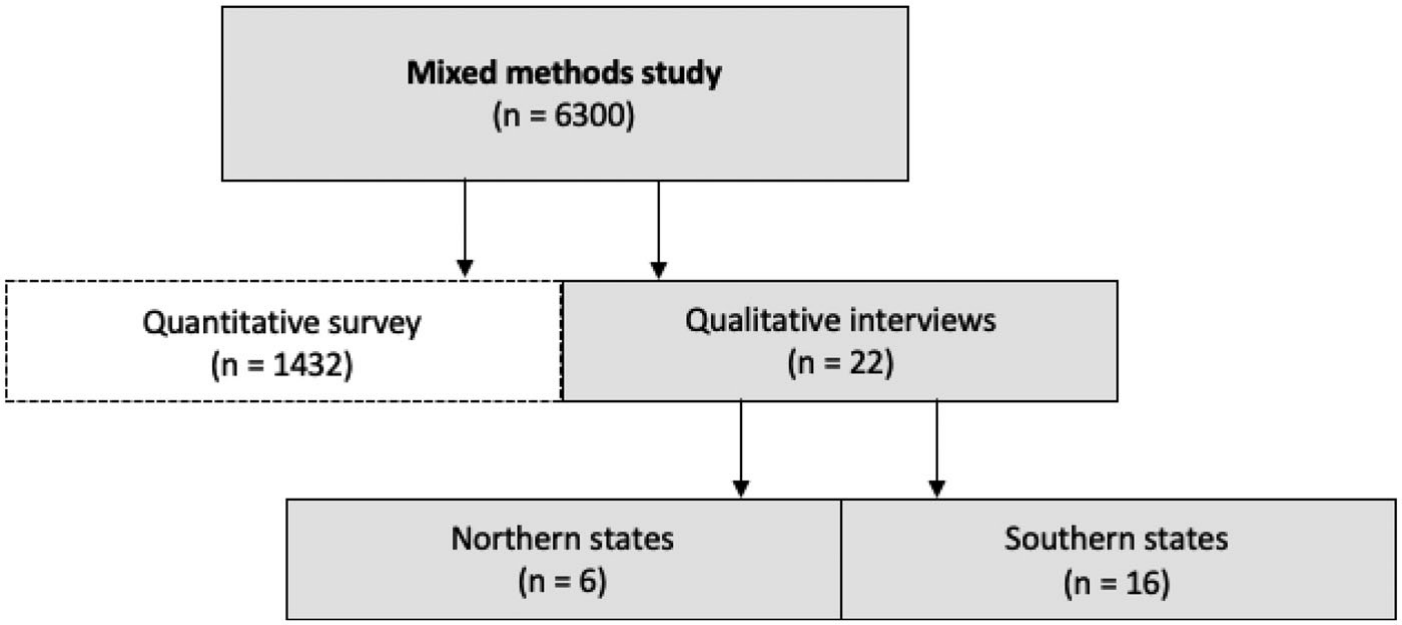
Flow chart illustrating the participants involved in the quantitative and qualitative part of the cross-sectional study with a mixed methods design; 6300 general practices in four federal states in Germany were randomly selected: 1980 practices in Schleswig-Holstein and Mecklenburg-Western Pomerania in the north, and 4320 practices in Bavaria and Baden-Wuerttemberg in the south of Germany. For feasibility reasons, states have not been randomised. The selected GPs represent approximately 14% of the German GPs [13]. Of the 6300 invited to participate in the survey and in the interview study, 1432 GPs participated in the quantitative part and 22 in the qualitative study. Of the 22 GPs interviewed, 6 were GPs from the north (3 from Mecklenburg-Western-Pomerania and 3 from Schleswig-Holstein) and 16 were GPs from the south of Germany (7 from Baden-Wuerttemberg and 9 from Bavaria). There were no dropouts in the qualitative study. Quantitative results are not reported in this article.

In Germany, 6300 general practices in four federal states were randomly selected by Arztdata, a commercial provider of address lists [10]. Two northern and two southern states were selected due to the number of COVID-19 cases and thus [11], the expected experience in practices at the onset of the pandemic differed substantially. The GPs were invited to participate in a cross-sectional survey and qualitative telephone interviews. One resident GP from each practice was eligible to participate in this qualitative study (convenience sample). Of the 6300 invited GPs, every participant who was interested was included until no or little change to the coding framework and no new issues emerged (data saturation) [12]. In total, 22 GPs participated in the interview study. A summary of the participants involved in the cross-sectional study is available as Figure 1.

### Data collection

The research team was multidisciplinary and consisted of practising clinicians, academic GPs and qualitative research experts. Interviews were conducted by one researcher (LM) trained and supervised by experienced qualitative researchers (IG, DG, HS, SP). All interviews were audio recorded and transcribed verbatim. Participants’ characteristics were independently collected without recording (Supplementary Material 1). The research team (IG, HK, SJ, HS, SP, LM) developed a semi-structured interview guide based on practising clinicians’ opinions and relevant qualitative studies examining GPs’ and healthcare workers’ experiences during the pandemic [4,14]. The interview guide was pilot tested in one interview. It was adapted to newly emerging themes during the interviews that required more data to understand the meaning fully [15,16]. Individual topics reaching saturation at an early point of data collection were not further explored in the interviews, as sufficient data had been gathered to understand the topic with its complexity and depth, for example, regarding financial consequences of the pandemic [16]. Identity, names, and other identifying details were anonymised during the transcription. The interviewer made field notes during the interviews. The core questions of the interview guide are presented in Table 1.

**Table 1.** Semi-structured interview guide.

Is there a topic you want to start with in terms of the COVID-19 pandemic or that comes to your mind immediately?	
1. Healthcare system Perspective on the healthcare system during the pandemic Subjective assessment of patient care Own thoughts for improving care	
Leading question/questions	Presentation options
What do you think of healthcare during the pandemic? Or: What did you notice about healthcare during the pandemic? Or: What has been your impression of healthcare during the pandemic? Or: What was your experience of healthcare during the pandemic?	How would you describe the organisation of primary care during the pandemic? How would you assess outpatient and inpatient care during the pandemic? What other positive and negative developments in dealing with the pandemic have you observed in the healthcare system? What are your wishes regarding care of COVID-19 patients? From your point of view, what are the biggest problems in dealing with the pandemic right now? In your view, what needs to be improved urgently in healthcare?
2. Practice organisation and employees/co-workers Impact of the pandemic on practice routines Impact of the pandemic on team members Adaptation strategies in the practice	
Leading question/questions	Presentation options
What has changed in your practice throughout the pandemic?	How have you been dealing with the pandemic in your practice? How has the pandemic affected the organisation of your practice? How has your staff reacted to the pandemic? What impact has the pandemic had on your work? To what extent has the pandemic affected your practice financially? How have you been dealing with this in your practice? What has been your experience in providing patient care during home visits?
3. Patient care Implications for treatment approaches Experiences with COVID-19 patients	
Leading question/questions	Presentation options
What impact has the pandemic had on your patient care?	To what extent has the treatment of non-COVID patients changed in the pandemic? What steps have you taken to care for your COVID-19 patients?
4. Personal implications Coping strategies Use of/ access to support resources Personal adaptation strategies Coping strategies with the disease in the professional environment (Mutual support? Being left alone? Denial of a risk?) Coping strategies with the disease in the private environment (support, stigmatisation of the person exposed to COVID-19?)	
Leading question/questions	Presentation options
How have you personally dealt with the pandemic?	What impact has the pandemic had on your private life? How has your private environment dealt with the pandemic? Did people in your environment get sick? What forms of support have you experienced during the pandemic? What has been helpful for coping with the pandemic? What impact has the pandemic had on your family life?
Would you like to add anything else?	

### Analysis

Data were analysed using Kuckartz’s inductive and deductive qualitative structuring content analysis [17], a well-established approach for managing qualitative data in healthcare research [18,19]. Data were managed using MAXQDA (version 2020). Deductive categories for the first coding cycle of eight interviews were created and thematic case summaries served as an intermediate step for analysis. Subcodes were formed deductively, derived from the research question and interview guide, and inductively in a subsequent procedure. The interviews were then analysed using the preliminary coding framework. To ensure intersubjective reproducibility [20], 30% of the interviews [21] were coded independently by three researchers (LM, HS, JEM).

The coding of the text passages was then compared and discussed until reaching internal consensus. In case of disagreement, the categories formed were discussed several times by the research team (IG, LM, DG, HS, SP) until agreement was achieved. In the following rounds of the analysis, the coding framework was developed further until the system of subcodes appeared saturated and no further subcodes needed to be redefined. The creation of codes took place simultaneously with data collection. Transcripts were not returned to the participants for comment or correction.

### Ethics

The study was approved by the Ethics Committee of the Medical Department at the Julius-Maximilians-University in Wuerzburg, Germany (reference number 135/20-am). All participants signed informed consent for the interviews to be audio recorded and transcribed. Informed consent was given by both email or mail. No compensation for participating was provided.

## Results

Of the 22 participants, 13 (59%) were males, 41% were aged 51–60 years, 64% reported working in a solo practice and 32% in a group practice. The participants’ demographics are shown in Table 2.

**Table 2.** Characteristics of participants.

Sex	Number	% (*n* = 22)	Participation in a COVID-19 service	Number	% (*n* = 22)
Female	9	41	Yes	12	55
Male	13	59	No	10	45
Age (years)	Number	% (*n* = 22)	Professional experience in primary care (years)	Number	% (*n* = 22)
31–40	2	9	<5	2	9
41–50	6	27	5–14	7	32
51–60	9	41	15–24	6	27
61–70	4	18	25–34	6	27
71–80	1	5	35–44	0	0
>80	0	0	45–54	1	5
			>55	0	0
Risk group^a^	Number	% (*n* = 22)	Risk group at home^b^	Number	% (*n* = 22)
Yes	8	36	Yes	8	36
No	14	64	No	14	64
Practice location^c^	Number	% (*n* = 22)	Federal state	Number	% (*n* = 22)
Rural	8	36	Baden-Wuerttemberg	7	32
Suburban	8	36	Bavaria	9	41
Town	3	14	Mecklenburg-Western-Pomerania	3	14
City	3	14	Schleswig-Holstein	3	14
Position	Number	% (*n* = 22)	Type of practice	Number	% (*n* = 22)
Employed GP	2	9	Solo	14	64
Self-employed GP in a practice	18	82	Group	7	32
Equally entitled GP in a group practice	2	9	Ambulatory care centre	1	4
			Practice with several locations	3	14
			Practice with one location	19	86
Number of GPs in practice	Number	% (*n* = 22)	Number of HCAs^d^ in practice	Number	% (*n* = 22)
1	12	55	1	0	0
2	5	23	2	6	27
3	3	14	3	3	14
4	0	0	4	5	23
5 or more	2	9	5 or more	8	36

^a^Response of participants whether they feel that they belong to the risk group for severe COVID-19.

^b^Response of participants whether they feel that their family home members belong to the risk group for severe COVID-19.

^c^Rural (<5.000 inhabitants), suburban (5.000–<20.000 inhabitants), town (20.00–100.000 inhabitants), city (>100.000 inhabitants).

^d^HCAs: healthcare assistants.

The following main themes were formed: Healthcare system-associated changes, changes in practice routines, changes in patient care, changes in personal life, and improving health crisis preparedness. The categories are presented in Table 3. Additional verbatims (V1-30) are available as Supplementary Material (Box 1).

**Table 3.** Summary of themes regarding GPs’ experiences of the COVID-19 pandemic.

Main codes	Subcodes
1. Healthcare system-associated changes	Coordination with healthcare authorities Support provided by the government Adaptation to the lack of equipment
2. Changes in practice routines	Financial impact Strategies to secure care New dimensions of teamwork
3. Changes in patient care	Uncertainties while providing care to COVID-19 patients Dynamics in the doctor-patient relationship
4. Changes in personal life	GPs’ understanding of their professional role Psychological distress
5. Improving health crisis preparedness	Improving crisis management Access to clear and consistent information Empowering people with crisis management experience Recognition and support of GPs’ efforts Stimulating social solidarity

### Theme 1: Healthcare system-associated changes

#### Coordination with healthcare authorities

Most participants criticised the inconsistency of pandemic regulations in Germany. Communication with health authorities was described as insufficient (V1). Furthermore, constantly changing measures, recommendations, and guidelines in primary care were reported as an additional burden. GPs described situations where patients were more up-to-date than their GP and perceived this as frustrating.

*[…] Patients suddenly call us, say they want to be tested. This had just been permitted and we didn’t even know that. […] it feels like we’re working on the frontlines, and we’re not even really supported. (GP20)*

Participants expressed that health departments had failed to adapt their capacities to the epidemiological situation. They reported that bureaucratic obstacles impeded a rapid response to the COVID-19 crisis and led to an increased workload in everyday practice, for example, due to complicated billing systems for smear tests or the expanded use of video consultations (V2).

*[…] I need just as much time for the swab test and explaining it as I do afterwards for processing [the swab test] and bureaucracy […]. It’s annoying. (GP9)*

#### Support provided by the government

Participants described a lack of sufficient protection, guidance, and support, even though they felt like playing a vital role in the pandemic response (V3). However, testing centres were perceived as a great relief and it was criticised that some of them were suddenly closed during the pandemic. In addition to crisis management, they also perceived that the government claims that GPs take on numerous further tasks for which they did not feel responsible.

*I’m mostly annoyed, that […] all the other testing centres […] have been closed and the whole pandemic [management] is completely assigned to GPs. I don’t think that GPs are responsible for overcoming […] pandemics, but we are responsible for the 49,999 other diseases that patients are dealing with and we have to answer for them and be there for those [patients]. (GP19)*

#### Adaptation to the lack of equipment

Participants mentioned that PPE was scarce and could only be purchased at elevated costs. However, they felt this resulted in a strong sense of solidarity among the population, as residents, friends and colleagues donated protective equipment (V4).

In addition, participants reported that they had become creative due to the shortages of critical healthcare supplies, for example, medical masks were heated in the oven to be re-used. Also, they would find new suppliers or make PPE themselves.

*[…] I ordered exam gloves from my hairdresser’s this week (laughs) because we have not been able to get any on the main market anymore and especially not in a normal price range. (GP14)*

### Theme 2: Changes in practice routines

#### Financial impact

The responses ranged from increased costs at the pandemic’s beginning to financial benefits (V5,6). However, financial losses were not perceived as an existential threat to the practice but rather as a temporary state, especially as there was a sharp increase in consultations after the first wave.

*[…] I’ve been in this business for 30 years […] I don’t struggle with this anymore. […] Of course, there are always certain fluctuations over time. That’s completely normal […]. (GP6)*

#### Strategies to secure care

Participants described their efforts and adaptive changes in their routines to ensure continuity of care, quickly implementing new infection control measures in their practices (V7–9). For example, special infection consultation hours were offered, infection areas were set up outside or additional isolation rooms were rented to separate patient streams. In addition, it was reported that shift systems were introduced, staff was divided to work in teams and cleaning and disinfection were enhanced. Also, telecommunication was used more frequently.

*I have video consultation hours, but I don’t use them regularly, but I use them for nursing home patients. That is, if there is something that I used to look at in the home, I now have it shown to me by camera. […] it works well. And it’s also accepted. (GP13)*

#### New dimensions of teamwork

Participants perceived teamwork in the practice as a particularly important source of support to solve problems during the COVID-19 outbreak (V10). Even though new tensions in the team were described as well (V11), most of the GPs reported that workplace communication improved, and team cohesion was strengthened during the pandemic. Also, sharing COVID-19-related practical challenges with the staff allowed new ways to solve problems together and produce innovative ideas adapting practice procedures.

*Well, it has probably even strengthened the team, as we’ve stuck together. Every day we had to improvise. I feel like it’s rather bound us together. (GP5)*

In this context, effective teamwork and professional collaboration were perceived as essential sources for emotional support and resources for overcoming frustration and the sense of overload caused by the pandemic (V12).

### Theme 3: Changes in patient care

#### Uncertainties while providing care to COVID-19 patients

Uncertainties in diagnosing COVID-19 were described due to the variety of symptoms and insufficient data on the clinical presentation (V13).

*I don’t understand why we don’t have real data of autopsies up to now or know more about the clinical features […]. It says everywhere, they [patients with COVID-19] don’t have a rhinitis. The ones that I saw, they all had a rhinitis, […] we just know too little. (GP18)*

#### Dynamics in the doctor-patient relationship

It was reported that patients were seeking more advice from their GP, as there was an increased need for reassurance, information, and emotional support. One important focus of the doctor-patient interaction was counselling, discussing COVID-19 and pandemic-related issues, as well as general health problems, concerns, and fears. In this context, patients would expect their GP to be the all-knowing doctor, which was experienced as burdensome (V14). Participants stressed that social distancing might affect their relationship of trust with their patients; for example, non-verbal and verbal communication was described as more challenging. Furthermore, wearing medical masks and protective clothes and the extensive distance rules were perceived as barriers to the patient (V15)

*[…] especially for a GP, this covering [with PPE] is, of course, damaging the relationship of trust with the patient, by showing how dangerous they might be […]. (GP19)*

Even though most of the patients were described as compliant with COVID-19 rules, participants reported that some patients increasingly expressed their discontentment about long waiting hours for a COVID-19 test result or the scarcity of flu vaccine doses (V16,17). Patients would develop a certain sense of entitlement, for example, they demanded to see their GP for an examination appointment as soon as possible without appreciating their GP’s efforts.

### Theme 4: Changes in personal life

#### GPs’ understanding of their professional role as physicians

Participants perceived themselves as important role models with a high responsibility for their patients, team, and family members (V18). In the interviews, they reported that a potential drop-out could hardly be managed by colleagues in surrounding practices.

*[…] I have a high responsibility to care for my patients. If I must take sick leave because of carelessness, 2.000 patients won’t be taken care of all of a sudden. (GP15)*

Especially in rural areas, participants reported difficulties in meeting the residents’ expectations towards their GP to be a role model also in one’s private life (V19).

#### Psychological distress

Most participants reported constraints of the pandemic in their private life (V20,21), such as the fear of infecting relatives or the absence of leisure activities to compensate for their stressful workday. However, some perceived the pandemic onset as an opportunity to spend more time with their family (V22), especially in the context of a temporary consultation decrease. Overall, participants could put their psychological stress into perspective given the private burdens reported by many patients.

*[…] my husband and I, we’re really in a position to teach our children […] at home and to support them. But we’re privileged and not many families are as lucky as we are. (GP4)*

Some GPs reported excessive working hours in everyday practice and felt overworked, strung up, and overburdened (V23).

### Theme 5: Improving health crisis preparedness

Participants suggested potential strategies to overcome the pandemic: They addressed the importance of improving crisis management through consistent and clear guidelines, fewer bureaucratic obstacles, and quick access to adequate PPE in future health crises (V24,25). Furthermore, the GPs perceived one official source of clear and consistent information, easy access to accurate information and sources for advice, and the promotion of collegial exchange as crucial (V26).

*[…] now, half a year later, as one already knows more about COVID-19, one could perhaps give practices some kind of a manual, […] what’s worked quite well at least in the outpatient sector for other colleagues in the treatment of symptoms. (GP4)*

Participants also highlighted the importance of empowering people with public health and crisis management experience to make crisis-response decisions (V27). Another suggestion for supporting GPs and expressing public appreciation of their contribution to the crisis was providing financial and human resources to support GPs, for example, strengthening research in primary care and promoting collaboration with the inpatient sector (V28,29). Lastly, participants wished to stimulate social solidarity, especially through reliable administrative information that creates awareness of the importance of infection control measures while not generating fear, so that pandemic restrictions can be accepted in the long-term (V30).

*[…] I’m grateful for any reasonable communication [with the population by authorities]. […] What I mean by that is: neither panicking nor trivialising but finding some kind of a middle ground. (GP12)*

## Discussion

### Main findings

This qualitative study gives insight into GPs’ perceptions and experiences in Germany during the first wave of the COVID-19 pandemic and identifies barriers and facilitators of infectious disease control. In the interviews, five themes were illuminated: Healthcare system-associated changes, changes in practice routines, changes in patient care, changes in personal life and improving health crisis preparedness. The respondents described that confusing and delayed communication and high administrative workload were mainly hindering quick and efficient practice management during the pandemic. However, participants managed to adapt their practice routines to the crisis rapidly. Teamwork and communication in the practice improved, which were important factors that helped GPs cope with their difficult working conditions. Even though respondents stated that care was successfully secured, they expressed their worries about social distancing in the practice impacting the therapeutic relationship negatively. In their professional and private lives, participants perceived themselves as important role models with a high sense of responsibility for others. To support GPs, the importance of clear and consistent guidance by health authorities and reliable information was highlighted.

### Strengths and limitations

Orienting towards Kuckartz’s qualitative structuring content analysis approach during the process of data collection, analysis and representation [17], we captured GPs’ perceptions from their subjective perspective, as they were directly affected by the pandemic. Semi-structured interviews allowed for data depth and a wide range of reported experience [22].

Several limitations in our study should be noted. First, some reported experiences might be distorted due to retrospective data collection, as the participants were asked to recall their experiences during the first months of the pandemic in addition to their current perceptions. To mitigate interviewer bias, the interviewer was trained in interview skills, data collection was supervised and a semi-structured interview guide was used. Second, as the participants represent a convenience sample, the possibility of selection effects should also be noted, for example, particularly motivated and resilient GPs or less stressed GPs might have taken part in this study. However, we interviewed GPs who varied in their characteristics (age, type of practice, geographically different parts of Germany), included participants until data saturation was reached, and reported contrasting viewpoints to mitigate potential selection effects. Lastly, it should be noted that this study was not designed to generalise our findings to other settings. The results are a snapshot and need to be interpreted in the light of the fast-moving progression of the pandemic.

### Interpretation

GPs played a key role in providing care to COVID-19 patients and had to restructure their practice management quickly. Participants’ adaptability and resourcefulness in implementing safety measures into their practices have also been reported in previous studies [4,23]. Furthermore, an increase in remote consultations to maintain contact with patients in light of transmission restrictions is in line with the literature [4,6]. The respondents described good self-management strategies, though this should be evaluated in light of the relatively low infection numbers during the interview period. Consistent with other studies [7,24], the participants in this study stated being overworked and overwhelmed, as they faced new forms of stress. Consequently, strengthening support systems and providing psychological counselling seems highly relevant in a prolonged crisis situation such as the COVID-19 pandemic to prevent exhaustion of medical staff [14].

In line with previous studies [4,8,24], administrative obstacles, lack of cooperation with health authorities, and inconsistent or delayed information presented major challenges for respondents in our study. On the contrary, generous compensation for financial losses was perceived as helpful. Also, testing centres, and other COVID-19-specialised outpatient practices, reduced pressure on GPs, which is consistent with previous literature [25]. Other studies have stated that outpatient physicians criticised crisis management more than their inpatient colleagues, which may indicate that the key role of outpatient care and specifically primary care, is not reflected in adequate support services [26].

The GPs in our study described themselves as vital role models with a high sense of responsibility. They rather expressed their concern about passing the virus onto others or absence due to infection than becoming infected and reported a high level of trust in the German healthcare system, as well as in their individual health status. It should be noted that none of the respondents had previously been ill or reported infections in their own family. Also, the participants had mainly observed mild disease courses in their patients. In a similar study, Belgian GPs were mainly concerned about transmitting the virus because they considered their own infection risk high [4].

In the interviews, concerns about the potential damaging impact of the pandemic on the doctor-patient relationship were expressed. In addition, deterioration in the therapeutic alliance during the pandemic was reported. A contrasting picture is presented in a similar study in Italy, describing a high level of appreciation and solidarity between GPs and their patients [3]. Reported uncertainties in diagnosing COVID-19 in our study due to a wide range of symptoms are confirmed in the literature [2].

Participants also reported an increased exchange with team members, which allowed for adopting more creative solutions for practice management. Promoting teamwork in practices seems particularly important as resilience is strengthened [27], and organisation and quality of care can be improved successfully [28].

### Implications

GPs played a key role in pandemic response by caring for most patients and reducing pressure on hospital care. Their experience and adaptive performance indicate that involving GPs and their teams in decision-making processes by health authorities maybe support the management of future pandemics. Fewer bureaucratic hurdles in implementing infectious disease control measures in practice routine and consistent information would ease the workload of the practice team. Information of the population in accordance with GPs’ information also seems relevant to stimulate protective behaviour and reduce pressure on primary care, for example, avoiding contradictory information and panic-mongering [29].

As this study is only an insight into GPs’ experiences during the first year of the pandemic, a follow-up study might be helpful to investigate GPs’ experiences during the pandemic over time [3], especially regarding the impact of social distancing on the doctor-patient relationship and the relevance of teambuilding in the primary care context.

## Conclusion

Patient care was seen as successful but bureaucratic obstacles and lack of communication limited GPs’ adaptation to the crisis. Participants wanted clear information and consistent involvement from health authorities. The results indicate that a robust primary care system is critical to effective COVID-19 management. They also underscore the importance of policymakers providing adequate support services to strengthen primary care in future health crises.

## Supplementary Material

Demographic questions

Box 1. Descriptions of categories and additional verbatims.

## Abbreviations

COVID-19: coronavirus disease 2019
DG: David Gierszewski
GP: general practitioner
HCAs: healthcare assistants
HK: Hanna Kaduszkiewicz
HS: Hanna Schrader
IG: Ildikó Gágyor
JEM: Jana Ehlers-Mondorf
LM: Lisa Makowski
PPE: personal protective equipment
SJ: Stefanie Joos
SP: Sandra Parisi.
