# Supplementary material for: German general practitioners’ experiences during the COVID-19 pandemic and how it affected their patient care: A qualitative study
Source: Eur J Gen Pract. 2023 Feb 1;29(2):2156498. doi: 10.1080/13814788.2022.2156498 (PMC10249445; doi:10.1080/13814788.2022.2156498)
Supplement: Demographic questions [file IGEN_A_2156498_SM1979.docx]

**Supplemental Material 1. Demographic questions***

| 1. What is your gender?  □ male  □ female  □ divers |
| --- |
| 2. How old are you?  □ 18-30 years  □ 31-40 years  □ 41-50 years  □ 51-60 years  □ 61-70 years  □ >80 years |
| 3. Do you think you belong to the risk group for severe Coronavirus disease?  □ yes  □ no  4. Do you think your family home members belong to the risk group for severe Coronavirus disease?  □ yes  □ no |
| 5. Where is your practice located?  □ rural (< 5.000 inhabitants)  □ suburban (5.000 - < 20.000 inhabitants)  □ town (20.00-100.000 inhabitants)  □ city (> 100.000 inhabitants) |
| 6. In which federal state is your practice located?  □ Baden-Wuerttemberg  □ Bavaria  □ Mecklenburg-Western-Pomerania  □ Schleswig-Holstein |
| 7. How many years have you worked as a GP?  □ <5 years  □ 5-14 years  □ 15-24 years  □ 25-34 years  □ 35-44 years  □ 45-54 years  □ >55 years |
| 8. What is your position in the practice?  □ employed GP  □ self-employed GP in a practice  □ equally entitled GP in a group practice |
| 9. What is the type of the practice? (solo/group and one location/several locations)  □ solo  □ group  □ ambulatory care centre  □ practice with several locations  □ practice with one location |
| 10. How many GPs work in the practice?  □ 1  □ 2  □ 3  □ 4  □ 5 or more |
| 11. How many HCAs** work in the practice?  □ 1  □ 2  □ 3  □ 4  □ 5 or more |
| 12. Are you participating in a COVID-19 service?  □ yes  □ no |
| *No audio recording to ensure anonymity of participants. |
| **HCAs = healthcare assistants |
