# Supplementary material for: German general practitioners’ experiences during the COVID-19 pandemic and how it affected their patient care: A qualitative study
Source: Eur J Gen Pract. 2023 Feb 1;29(2):2156498. doi: 10.1080/13814788.2022.2156498 (PMC10249445; doi:10.1080/13814788.2022.2156498)
Supplement: Box 1. Descriptions of categories and additional verbatims. [file IGEN_A_2156498_SM1801.docx]

| **Box 1. Descriptions of categories and additional verbatims.** | | |
| --- | --- | --- |
| **Main codes** | **Subcodes** | **Verbatims** |
| Healthcare system associated changes | Coordination with healthcare authorities | V1: ‘(sighs) Well, we don’t hear anything from the local public health department. So I think I’d rather not call them, as I guess, we won’t come through anyway.’ (GP9)  V2: ‘Well, that’s rather more the organisation during the pandemic, which has burdened us, than the pandemic itself [...].’ (GP1) |
|  | Support provided by the government | V3: ‘Sometimes you have that feeling [...] that they [the government] don’t work with us, with the general practitioners, even though we manage the primary care of the population, and that, that you’re left alone [...].’ (GP20) |
|  | Adaptation to the lack of equipment | V4: ‘Then, the face masks, [...] there was a call, [...] it was a gynaecologicst, [...] he had a few [masks] left, if I wanted to have a few more.’ (GP22) |
| Changes in practice routines | Financial impact | V5: ‘[...] I’m supposed to pay ten times more than the regular price for a FFP2 mask. This can be quite upsetting.’ (GP8)  V6: ‘We’ve gained financially from the pandemic [...], there was even a slightly higher number of cases than in the previous quarters [...], whereas for several practices, the practice was closed up for two weeks due to quarantine or infection of staff and we covered them [providing extra financial gain] [...].’ (GP13) |
|  | Strategies to secure care | V7: ‘I actually painted signs myself that masks are compulsory in the practice here, and for everyone – both, patients and stuff. [...] I’ve made a spit protection wall myself with plastic sheets of some old calendars [...].’ (GP12)  V8: ‘[...] how do you organise it with these smear tests in the practice? In rural areas, maybe it’s working. In the city [...] it’s really not that funny. [...] we’ve sometimes done it like this, that they [the patients] have to go on the balcony [...].’ (GP20)  V9: ‘And for the Corona-patients or patients with a cold, there is a tent in front of the practice, a big house tent, to take smear tests there, examining potentially infectious patients [...] That’s possible because we have parking spaces in front of the practice.’ (GP21) |
|  | New dimensions of teamwork | V10: ‘What’s really helpful is that I work together with a really great colleague now [...] I think that would have driven me crazy if I’d always had to somehow decide and sort out everything on my own.’ (GP14)  V11: ‘It’s a bit difficult within the team. We certainly have employees who take it very seriously and are also careful. And we also have employees who are annoyed by the whole thing. And they also believe these conspiracy theories a bit [...].’ (GP15)  V12: ‘[...] my staff gave me a lot of support. [...] we often did rounds and talked about how we can best solve things [...]. My staff was great.’ (GP17) |
| Changes in patient care | Uncertainties while providing care to COVID-19 patients | V13: ‘[...] every patient could be potentially infectious [...], as nearly all my positive patients didn’t have a cold but came because of every possible other symptoms, such as foot pain, back pain [...].’ (GP16) |
|  | Dynamics in the doctor-patient relationship | V14: ‘It’s just unbelievable, [...] what we’re expected to look up: where a patient has come from, whether that was a risk area last week [...] the patients somehow always think: ‘Well, the doctor, he knows that.’ That’s somehow out of proportion.’ (GP14)  V15: ‘Before Corona, doctor-patient contact was more relaxed, it was friendlier, it was more informal. [...] The communication – also non-verbal – was simply better. Now, everything is blocked by masks [...] It has become more restricted.’ (GP22)  V16 ‘[...] when you have substitute patients, who don’t feel like sticking to anything, that’s always a bit difficult, but I’d say that 98% of the patients are very compliant, are totally understanding [...].’ (GP4)  V17: ‘The patients are insecure, the atmosphere is now especially – at the beginning it was rather only uncertain – now, it’s rather also aggressive or yes, unpleasant.’ (GP20) |
| Changes in personal life | GPs’ understanding of their professional role | V18: ‘So, I’ve completely cut off contact with my family home [...] So, I deliberately took myself out off the situation that I could possibly infect my family [...].’ (GP17)  V19: ‘[...] it was recommended to reduce alpine sports [...] and with the ski on my back, I’m visible and I thought: ‘What might the people think?’ I’m aware of my actions but I kind of act against the recommendations, which didn’t make sense in my opinion. This was really difficult for me.’ (GP1) |
|  | Psychological distress | V20: ‘I’m incredibly worried that I’ll infect my daughter or my husband [...]. That’s actually my main motivation to wear the mask even though, the patients are outside [of the practice] and only my assistants are around.’ (GP14)  V21: ‘So, for me, personally, it was intense because my partner no longer visited me because I’m a doctor, I was also attacked as a doctor, [...] I found that very horrible as I’d read before, that in Spain, the houses were smeared and that doctors should move out because they’d be the worst superspreaders [...].’ (GP5)  V22: ‘[...] one could see each other [family members] regularly, one could have lunch together, one could see the child on a regular basis, one could do a lot of sports.’ (GP13)  V23: ‘At some point, I think my brain was overloaded [...] and I couldn’t even remember the simplest of drug names anymore.’ (GP11) |
| Improving health crisis preparedness | Improving crisis management | V24: ‘So, complete extrication of general practitioners from the, from testing [for COVID-19]. That should fall within public health’s competence.’ (GP19)  V25: ‘[…] production of protective equipment. That simply belongs in the EU […] we have a CE mark and we must be able, if we produce the material as well as the filters in Germany, to also produce the masks here. And these are things that are of national interest.’ (GP15) |
|  | Access to clear and consistent information | V26: ‘[…] we’re looking for an air exchanger. That’s also something where we’re on our own, thinking: Isn’t there anybody who can tell us what to do or what’s a reasonable price range?’ (GP9) |
|  | Empowering people with crisis management experience | V27: ‘[…] I know that one friend of mine, she’s a general practitioner in X. They also had a doctor in charge for crisis management who had a lot of experience because he was somehow with the military in Afghanistan. […] if the situation should somehow worsen again, […] one simply has to put competent people in the right positions.’ (GP1) |
|  | Recognition and support of GPs‘ efforts | V28: ‘[…] but if politicians, […] take into account that we were actually important, there comes nothing. That’s why research is important. That one has something that the minister or his successor might read […] that we’re actually involved […].’ (GP8)  V29: ‘But for me, it’s simply about that the sectors - outpatient and inpatient – don’t patronise or even fight each other, but that they both should be willing to learn from each other and open their minds to what someone from the outside has to say to the hospital. Just as I open my mind to what someone from the hospital has to say to me.’ (GP2) |
|  | Stimulating social solidarity | V30: ‘[…] stricter measures, other measures, that’s all actually more or less nonesense in my view. It does only make sense if the population lives it. And if they live it and adapt their daily lives, […] we can all live a reasonable life.’ (GP16) |
